# A deep learning model for rapid classification of tea coal disease

**DOI:** 10.1186/s13007-023-01074-2

**Published:** 2023-09-09

**Authors:** Yang Xu, Yilin Mao, He Li, Litao Sun, Shuangshuang Wang, Xiaojiang Li, Jiazhi Shen, Xinyue Yin, Kai Fan, Zhaotang Ding, Yu Wang

**Affiliations:** 1 Tea Research Institute, Qingdao Agricultural University, Qingdao, 266109 China; 2 Tea Research Institute, Shandong Academy of Agricultural Sciences, Jinan, 250100 China

**Keywords:** Tea coal disease, RGB, Hyperspectral, Machine learning, Deep learning, Classification

## Abstract

**Background:**

The common tea tree disease known as “tea coal disease” (*Neocapnodium theae* Hara) can have a negative impact on tea yield and quality. The majority of conventional approaches for identifying tea coal disease rely on observation with the human naked eye, which is labor- and time-intensive and frequently influenced by subjective factors. The present study developed a deep learning model based on RGB and hyperspectral images for tea coal disease rapid classification.

**Results:**

Both RGB and hyperspectral could be used for classifying tea coal disease. The accuracy of the classification models established by RGB imaging using ResNet18, VGG16, AlexNet, WT-ResNet18, WT-VGG16, and WT-AlexNet was 60%, 58%, 52%, 70%, 64%, and 57%, respectively, and the optimal classification model for RGB was the WT-ResNet18. The accuracy of the classification models established by hyperspectral imaging using UVE-LSTM, CARS-LSTM, NONE-LSTM, UVE-SVM, CARS-SVM, and NONE-SVM was 80%, 95%, 90%, 61%, 77%, and 65%, respectively, and the optimal classification model for hyperspectral was the CARS-LSTM, which was superior to the model based on RGB imaging.

**Conclusions:**

This study revealed the classification potential of tea coal disease based on RGB and hyperspectral imaging, which can provide an accurate, non-destructive, and efficient classification method for monitoring tea coal disease.

## Introduction

Tea coal disease (*Neocapnodium theae* Hara) is a disease that occurs in tea and is caused by the presence of *Neocapnodium theae* Hara, or insects such as whitefly, scale, and aphid. It causes the leaves to wither and die. At the initial stage of the disease, small black circular or irregular spots appear on the surface of the leaves, which gradually expand. In extreme circumstances, the leaves become completely covered in black powdered coal, which then spreads to the twigs and stems. The surface of each part of the diseased plant is covered with a layer of bituminous coal [1]. Tea coal disease is widely distributed and occurs in various tea-producing provinces in China. When the disease occurs seriously, the tea garden presents a black and dirty area, and the growth of buds and leaves is hindered, resulting in a significant decline in tea production and a certain impact on tea quality [2]. Therefore, accurate, non-destructive, and efficient detection methods for tea coal disease are crucial for disease control.

Traditional diagnostic methods for tea coal disease, such as artificial visual surveys based on on-site disease symptoms, are prone to subjective influence and rely on professional knowledge. The similarity of symptoms can lead to misdiagnosis, which is time-consuming and laborious. Another method, physical and chemical experiments to detect crop diseases, is relatively objective and accurate, but it is costly, inefficient, and destructive to the tea plant itself [3, 4]. An authentic, efficient, and non-destructive testing method is needed to detect and control diseases on time.

At present, the detection of plant diseases and pests based on image processing and computer vision has become an important research direction. Non-invasive sensing technologies such as RGB imaging, thermal imaging, multispectral, and hyperspectral imaging are potential non-invasive tools for detecting agricultural plant diseases, with multiple advantages compared to traditional methods [5–7]. Although RGB images have only three bands of red, green, and blue information, and their recognition ability is limited, RGB cameras are portable and low-cost. Although hyperspectral images have hundreds of continuous bands, not only limited to the visible light portion of the spectrum but also have large amounts of information and high accuracy, their costs are high. Due to the integration of spectrum and image, hyperspectral imaging technology has shown significant advantages in providing objective, accurate, non-destructive, and intuitive plant disease diagnosis results. And hyperspectral imaging detection technology obtains disease image and spectral information without causing damage to crops.

Currently, RGB is widely used in other crop diseases. For example, Jayapal et al. [8] proposed a combined RGB image and deep learning approach for identifying root rot. Comparing the performance of this model with the Transfer learning model, this model achieved an F1 score of 88% and an accuracy of 89% in less inference time. Amarasingam et al. [9] proposed a method for detecting sugarcane white leaf disease by combining RGB images and deep learning based on UAV, evaluating the performance of existing deep learning models such as YOLOv5, YOLOR, DETR, and Faster R-CNN in sugarcane white leaf disease identification. The results showed that the YOLOv5 network had the highest accuracy, recall, and average precision of 95%, 92%, and 93%, respectively. Hallau et al. [10] proposed an algorithm for sugar beet leaf disease recognition based on RGB images captured from smartphone cameras. A support vector machine with a radial basis function kernel was used to classify the diseases. The results showed that the correct rate of classification for white leaf spot, Ramularia leaf spot, Phoma leaf spot, beet rust, and bacterial blight was 82%. Sie EK et al. [11] proposed a combined RGB image and mixed linear model approach to assess peanut leaf spot disease (LSD) and yield in West Africa. The results showed the effectiveness of the RGB image method as a high-throughput phenotyping tool for peanut LSD and yield assessment. Memon et al. [12] proposed a combined RGB image and deep learning approach to recognize cotton leaf disease, evaluating the performance of deep learning models such as custom CNN, VGG16, ResNet50, and the Meta Deep Learning model in the cotton leaf disease recognition. The results showed that the Meta Deep Learning model network has the highest accuracy of 98.53%.

Currently, hyperspectral imaging has applications in disease monitoring of other crops. For example, Feng et al. [13] used hyperspectral imaging technology (HSI) to detect leaf diseases in four rice varieties, and used a self-designed convolutional neural network (CNN) as the fundamental network for deep transfer learning methods. The accuracy of the three deep transfer learning methods exceeded 88%. Zhao et al. [14] identified the disease severity of wheat leaves infected with powdery mildew based on hyperspectral images and image segmentation techniques. A technical procedure for identifying and evaluating leaf-scale wheat powdery mildew was proposed. The results show that the SVM model constructed by PCA dimensionality reduction has the best effect, with a classification accuracy of 93.33%. Abdulridha, Batuman and Ampatzidis [15] used hyperspectral imaging (HSI) to detect citrus canker disease. The overall classification accuracy of the two classification methods, SVM with RBF kernel and KNN, were (94%, 96%, and 100%) and (94%, 95%, and 96%), respectively. Citrus crop infected with late-stage canker disease were successfully distinguished, with a classification accuracy of 92%. Wu et al. [16] used hyperspectral imaging combined with machine learning methods for strawberry gray mold identification and compared the accuracy of three machine learning models, Extreme Learning Machine (ELM), Support Vector Machine (SVM), and K-Nearest Neighbor (KNN). The results showed that the ELM classification model performed the best for classifying strawberry gray mold with an accuracy of 96.67%. Lee et al. [17] used hyperspectral imaging combined with machine learning methods to detect Basal Stem Rot (BSR) disease early in oil palm trees and compared the accuracies of multiple machine learning models. The results showed that all machine learning algorithms could isolate the infection stage, with overall accuracies of 86.67%, 66.67%, and 73.33% for MLP, SVM, and 1D CNN models, respectively. Currently, modeling algorithms are mainly divided into machine learning and deep learning. Among them, machine learning models not only achieve stable detection of crop diseases, but also demonstrate the ability to identify different stages of development of the same disease, leading crop disease detection technology to precision agriculture and intelligent agriculture. For example, Zhang et al. [18] collected hyperspectral imaging technology to obtain spectral information in the 384 to 1034 nm wavelength range of rapeseed sclerotinia sclerotiorum, evaluated the SSR detection index of diseased leaves using linear discriminant analysis, and established a partial least squares disease identification model with an accuracy of up to 85%. Harakannanavar et al. [19] proposed a tomato leaf disease detection method based on machine learning and image processing. Machine learning methods, such as SVM, K-NN, and CNN, were used and the accuracy of the proposed model was 88%, 97%, and 99.6%, respectively. Koc et al. [20] proposed a machine learning based method for the prediction of yellow rust in wheat. Using a machine learning approach (RF) combined with SVI data from spectral sensors in the RF model, the model prediction accuracy obtained was 0.50–0.61. Dias et al. [21] proposed a combined machine learning and UAV multispectral imagery approach to assess the severity of tomato late blight, and developed a random forest (RF) model to predict disease severity with a coefficient of determination of up to 0.93 for the test set.

However, deep learning has attracted much attention due to its outstanding performance in different artificial intelligence applications, and is widely used in computer vision [22]. Deep learning is a new field in machine learning research. Deep learning is a new field in machine learning research and is a method in machine learning based on learning representations of data as a branch of machine learning that is more effective. For example, Sujatha et al. [23] compared the performance of machine learning (SVM, RF, and SGD) and deep learning (Inception-v3, VGG16, VGG19) in detecting citrus plant diseases. The results show that deep learning methods perform better than machine learning methods: RF-76.8%<SGD-86.5%<SVM-87%<VGG19-87.4%<Inception-v3-89%<VGG16-89.5%. Ma et al. [24] compared the performance of machine learning (SVM, RF) and deep learning (DCNN) in recognition of four cucumber leaf disease symptoms. The results showed that the deep learning method DCNN had the best recognition with an accuracy of 93.4%. Wang, Sun and Wang [25] evaluated the performance of apple disease severity using pre-trained deep learning models based on transfer learning (e.g., VGG16, VGG19, Inception-v3, and ResNet50). Among them, VGG16 was the most high-performing model with an accuracy of 90.4%. Gao et al. [26] compared the performance of machine learning algorithm (SVM) and deep learning algorithms (XGBoost, and KNN) in recognizing wheat fusarium head blight. The results showed that the deep learning algorithm XGBoost had the highest performance with an accuracy of 93.63%. Sood et al. [27] used a deep learning based CNN transfer learning model for early recognition and classification of wheat rust on CGIAR image dataset. The results showed that VGG16 achieved 99.54% classification accuracy. However, there are no reports on the high-throughput acquisition and deep learning algorithms for spectral imaging data of tea coal disease infestation.

**Fig. 1:**
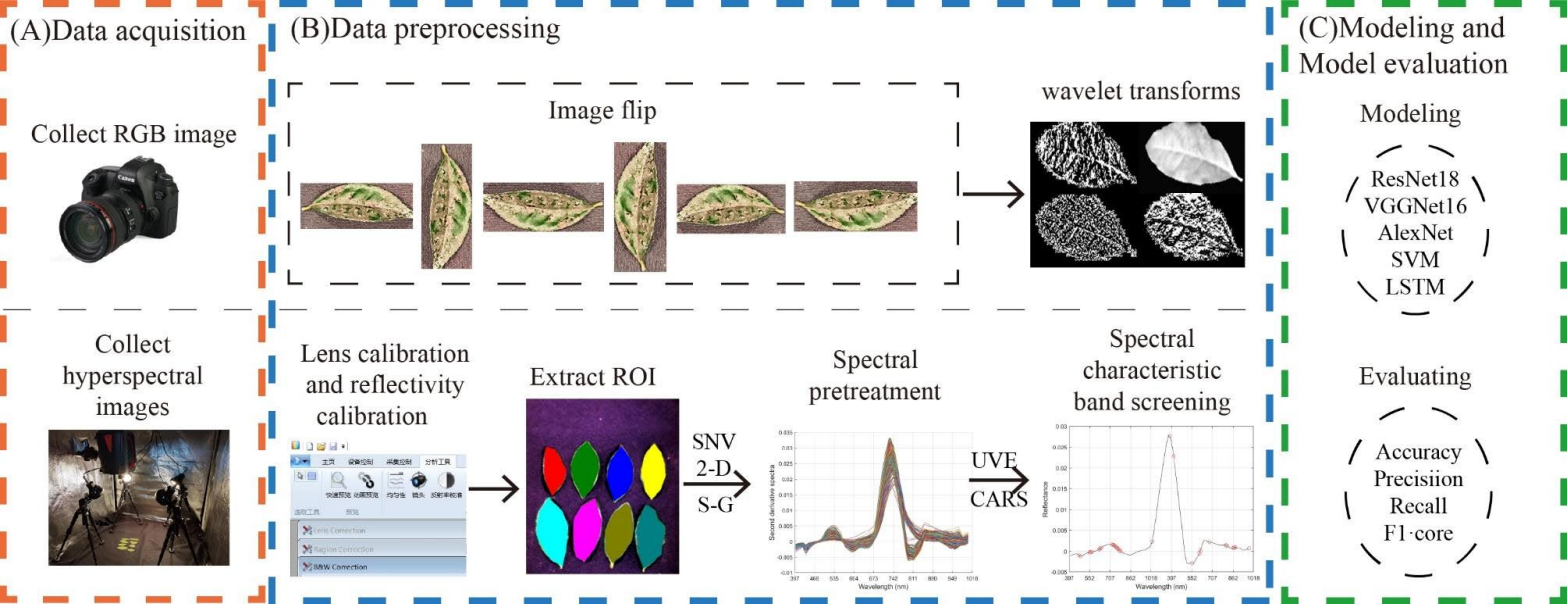
The overall framework of this study. (**A**) Data acquisition; (**B**) Data preprocessing; (**C**) Modeling and Model evaluation

This study collected tea leaves with different disease levels and obtained RGB and hyperspectral images. For the RGB images, they were flipped 90°, 180°, 270°, vertically, and horizontally to expand the sampled data by five times, followed by wavelet transform enhancement; for the hyperspectral images, the standard normal variation (SNV), the second derivative (2-D), and the Savitzky-Golay (S-G) algorithms were used to preprocess the spectral data, The CARS and UVE algorithms were used to filter the characteristic bands of the spectral data. A classification model for the severity of tea coal disease was established using deep learning algorithms such as ResNet18, VGG16, and AlexNet for RGB image data. A classification model for the severity of tea coal disease was established using SVM and LSTM algorithms for hyperspectral image data, and the model was evaluated using four metrics. This work demonstrated the potential of RGB and hyperspectral imaging-based tea coal disease classification, which could provide an accurate, non-destructive, and efficient way for tea coal disease monitoring. The general framework of this study is shown in Fig. 1. The main contributions of this study are as follows:

1. Comparison of two imaging techniques, RGB and hyperspectral.
2. Comparison of the optimization effect of two filtering feature banding methods, CARS and UVE, on the prediction model.
3. Comparison of modeling results with and without two-dimensional discrete wavelet transforms (2DWT) enhancement techniques.
4. Discussion of the prediction ability of machine learning and deep learning models such as ResNet18, VGG16, AlexNet, SVM, and LSTM.

## Materials and methods

### Study area

**Fig. 2:**
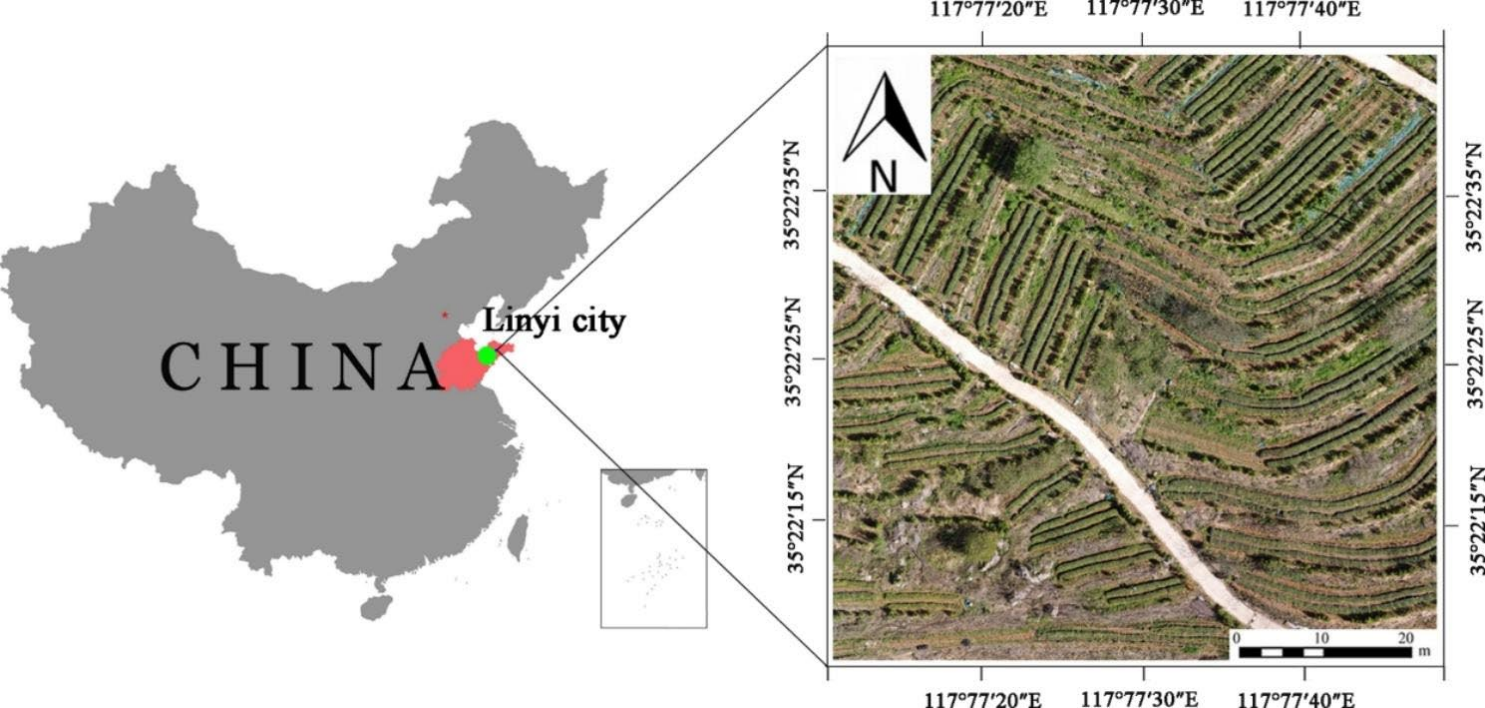
Location of experimental area

The experiment was conducted at Chunxi Tea Garden in Feixian County, Linyi City, Shandong Province, China (117°77’E, 35°22’N). The region has a warm-temperate monsoon continental climate with abundant light, four distinct seasons, mild climate and abundant rainfall, with an average annual temperature of 13℃, average annual sunshine hours of 2400–2600 h, and average annual precipitation of about 800 mm. The area of the test tea garden is more than 300 mu. The soil has a bulk density of 1.50 g cm-3, an organic matter content of 1.65%, and a pH of 5.8. The tea varieties planted in this tea garden include Zhongcha 108, Longjing 43, Jiukeng, and Longjingchangye. The location diagram of the test area is shown in Fig. 2.

### Data acquisition

This study was conducted in two seasons (spring and autumn) when tea plants in multiple plots of the Chunxi Tea Garden exhibited obvious symptoms of tea coal disease. Autumn was the first sampling, and about 250 tea leaf samples were randomly collected; the following spring was the second sampling, with about 400 tea leaf samples randomly collected. Experts are also invited to classify the collected leaves into four grades based on the severity of the disease: standard, mild degree, moderate degree, and severe degree. RGB and hyperspectral data were collected simultaneously for leaves with different disease levels.

The RGB image data were collected under natural light conditions using a digital camera (EOS·6D, Canon Co. Ltd, Beijing, China) with a picture resolution of (5184 pixels × 3456 pixels), a total of 700 pictures were taken, and the image storage format was JPEG, and the shooting angle was vertical. The RGB image data were flipped 90°, 180°, 270°, vertically, and horizontally to expand the sample data by a factor of 5.

Hyperspectral image data acquisition and calibration were performed according to the method of Huang et al. [28] Hyperspectral image data were acquired using a hyperspectral camera (GaiaField-Pro-V10, Jiangsu Dualix Spectral Image Technology Co. Ltd, China) in a built cube dark box. The data were lens-corrected and reflectance-corrected using the analysis tools of the data preprocessing software SpecVIEW (Jiangsu Dualix Spectral Image Technology Co. Ltd, China). The hyperspectral images in preprocessed RAW format were then opened with ENVI 5.3 (Research System Inc, Boulder, CO, USA), and the entire leaf sample was selected as the region of interest (ROI), and the mean reflectance spectral values of the samples were extracted using the ROI tool, and the spectral reflectance curves were saved to obtain a total spectral matrix of 650 × 176 (number of samples × number of variables) for data analysis.

### Hyperspectral equipment

**Fig. 3:**
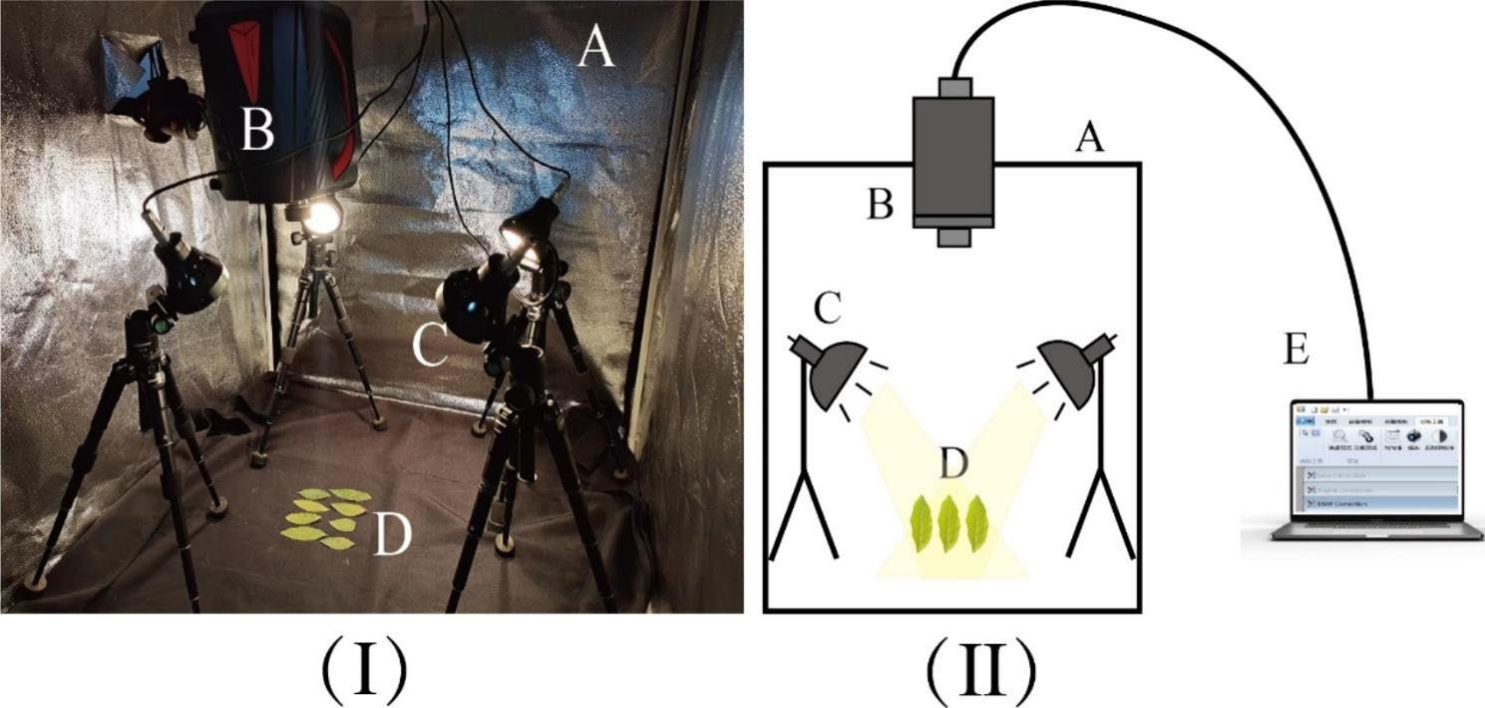
(**I**) Hyperspectral equipment real view. (**II**) Hyperspectral equipment mode diagram. (**A**) cube dark box; (**B**) hyperspectral camera); (**C**) 200 W halogen linear light sources; (**D**) tea leaf samples; (**E**) computer

The hyperspectral equipment used in this study is composed of an external cube dark box and an internal hyperspectral camera, a camera holder, four symmetrically distributed 200 W adjustable halogen linear light sources (hsia-ls-t-200w, China), an external computer, and other components. Place black flannelette under the leaf sample to ensure it is not affected by other reflective light sources. The parameters of the hyperspectral imaging system are set as follows: The model is GaiaField Pro-V10, and the hyperspectral camera has a 1936 × 1456 (space × Spectral) pixel, the spectral range of the captured image is in the visible and near-infrared band (400-1000 nm), and the reflectivity of 176 bands can be measured. The imaging method is a built-in push scan, with a spectral resolution of 3.5 nm, a frame rate of 7s/cube, a data interface of USB2.0, and a weight of 3 kg. The hyperspectral imaging device is shown in Fig. 3.

### Data preprocessing

#### Two-dimensional discrete wavelet transforms (2DWT) of varying degrees of disease images

**Fig. 4:**
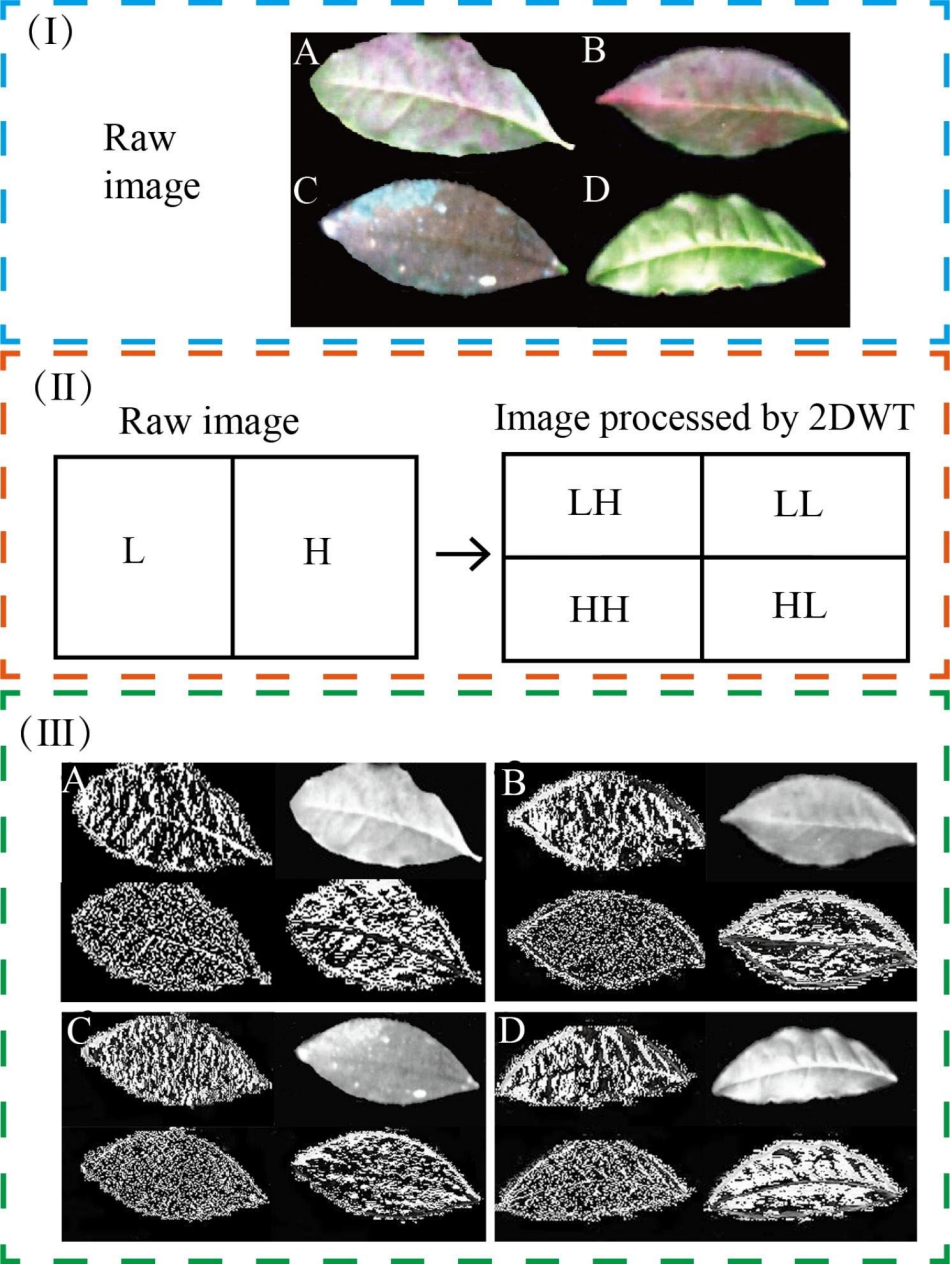
(**I**) Original image. (**II**) Schematic diagram of wavelet transform. (**III**) Leaf images with different degrees of disease after wavelet transform processing. (**A**) mild degree; (**B**) moderate degree; (**C**) severe degree; (**D**) standard

To better extract the feature information of the leaves of tea coal disease, the images of the diseased leaves were enhanced by wavelet transform. [29]This is because the wavelet transform enhancement processing applied in previous studies has made good progress in improving model accuracy, and the model has strong generalization ability and is suitable for tea tree disease classification. Wavelet transform can reduce or remove the correlation between different features of the extracted diseased leaf images by selecting appropriate filters. The RGB images of leaves with different degrees of disease are shown in Fig. 4 (I). The wavelet transform converts the image into a signal and then separates the signal in terms of low and high frequencies to obtain four components as shown in Fig. 4 (II). Where the LL component represents the low-frequency information of the image, the HL component represents the high-frequency information in the horizontal direction of the image, the LH component represents the high-frequency information in the multiply straight direction of the image, and the HH component represents the high-frequency information in the diagonal of the image. The leaf images with different degrees of disease after wavelet transform processing are shown in Fig. 4 (III).

During the decomposition of the image by wavelet transform, the LL component can be looped several times until the requirement is satisfied. In this paper, the LL component is looped only once for the discrete wavelet transform of an image f(x, y) of size A×B, as shown in Eq. (1):

1 $$W\phi \left(j_{0}, a, b \right)=\frac{1}{\sqrt{AB}}\sum _{x=0}^{A-1}\sum _{y=0}^{B-1} f \left(x, y\right){\phi }_{j_{0}, a, b}(x, y)$$

#### Spectral preprocessing

Spectral data is susceptible to interference from unwanted signals, which affects the modeling effect. Therefore, spectral preprocessing is required before data analysis to improve the accuracy and reliability of the model [30, 31]. The standard normal variation (SNV) can correct spectral errors caused by scattering. By preprocessing each spectrum, the spectrum is made as free of scattering error effects as possible. By centering and normalization, factors such as noise and signal shift in the spectral data can be eliminated so that different spectral data have the same scale as each other, improving the stability, reliability, and comparability of the spectral data. When processing a large amount of spectral data, centralization, and standardization can improve the efficiency and accuracy of data processing and reduce the processing complexity. The derivative processing can eliminate the impact of factors such as instrument background or baseline drift on the signal during data acquisition, resolve overlapping peaks, and improve resolution and sensitivity, but it also introduces some errors in the process. The second derivative (2-D) processing can eliminate the linear background shift and improve the spectral resolution and sensitivity. The Savitzky-Golay (S-G) method can effectively enhance the signal to noise ratio of spectral images and reduce the impact of random noise [32, 33]. It is a polynomial decomposition of the data within the moving window of the original spectrum using polynomials and fitting the data with least squares, which is essentially a weighted average method. This method is more straightforward and faster than similar smoothing methods, preserving distribution properties such as relative maxima, minima, and widths. Therefore, before modeling, we combined SNV, 2-D, and S-G preprocessing algorithms to preprocess the original spectral data of the sample.

#### Screening of characteristic bands

Screening characteristic bands is a commonly used technical means for spectral data analysis. Compared to other methods, characteristic band selection only selects the band related to target information from the full spectrum band and does not change the physical information of the spectrum [34]. The uninformative variable elimination (UVE) algorithm can remove the wavelength variables that are less efficient for modeling coefficients and select the characteristic wavelength variables, and the removed wavelength variables we call uninformative variables. The uninformative variable removal algorithm is based on the partial least squares (PLS) algorithm. Removing uninformative variables reduces the number of variables used for modeling and the model complexity. To select the uninformative variables, the UVE algorithm adds a set of white noise variables to the PLS model with the same number of variables as the original, and then obtains the regression coefficients corresponding to each variable, including the noise variables, based on the intersection and retention method of the PLS model [35, 36]. The stable values of each variable coefficient are divided by the standard deviation, and their quotients are compared with the stable values obtained from the random variable matrix to remove those wavelength variables that are as invalid for modeling as the random variables. The competitive adaptive reweighted sampling (CARS) is a feature variable selection method that combines Markov Chain Monte Carlo (MCMC) and PLS model regression coefficients, imitating the principle of “survival of the fittest” in Darwin’s theory. Then, the PLS model is built based on the new subset, and the wavelength in the subset with the smallest root mean square error of PLS model cross-validation is selected as the characteristic wavelength after multiple calculations [37, 38]. Therefore, before modeling, two feature band selection algorithms, UVE and CARS, are combined to analyze hyperspectral data. The basic parameters of CARS and UVE algorithms used in this study were shown in Table 1.

**Table 1:** The parameters of feature band selection algorithms

Algorithm	Parameters	Value
CARS	Method	None
	Fold	10
	Number of PCA	10
	Monte Carlo sampling times	300
UVE	Optimal factor number	5
	Leave-One-Out	700
	Cutoff	0.99

### Modeling and model evaluation

#### Modeling

Hyperspectral image data is modeled using SVM and LSTM algorithms. The SVM is a nonparametric machine learning method based on statistical learning theory and structural risk minimization [39, 40]. It can maximize separation or edges between different categories of samples by constructing a set of hyperplanes. It has several unique advantages in solving small sample, nonlinear, and high-dimensional pattern recognition problems. The phenomenon of " Curse of Dimensionality” and “Over learning” can be largely avoided. Several parameters should be evaluated and specified, including kernel functions, gamma values, and costs [14].The LSTM model can better process sequential data [41]. The architecture of the LSTM model consists of a LSTM Layer with 20 NumHiddenUnits, two FullyConnected Layers, a Softmax Layer and a Classification Layer [42]. It is a recently popular recursive neural network in machine learning [43]. Designed to avoid the problem of long-term dependencies, it has proven to be very effective in capturing long-term dependencies.

The RGB image data is modeled using deep learning algorithms such as ResNet18, VGG16, and AlexNet. The basic architecture of the ResNet 18 network is ResNet, and the depth of the network is 18 layers, including the convolutional layer and fully connected layer, excluding the pooling layer and Batch Norm layer. When the network is “not too deep,“ it simplifies optimization by providing faster convergence at an early stage. Moreover, the residual structure can accelerate learning, make the model easier to learn, and effectively prevent the exploding gradient problem or vanishing gradient problem. The ResNet18 network model is more accurate than the primary network model. VGG16 includes 13 convolutional layers, 3 fully connected layers, and 5 pooling layers. The model is relatively stable and easy to transplant. The most prominent feature is its simplicity, as the entire network uses the same convolutional kernel size (3 × 3) and maximum pool size (2 × 2). Convolutional concatenation has fewer parameters and more nonlinear transformations than a larger convolution kernel alone. And convolution kernels are concatenated to extract features multiple times, which is more delicate than single convolution kernels. The AlexNet model, which uses Relu for the first time and conducts multi GPU training, dramatically reduces the amount of computation and accelerates the convergence speed. To prevent overfitting and improve generalization capabilities, overlap pooling, data enhancement, and the introduction of dropout are implemented. The validation set was used for parameter tuning. The neural network parameters with the highest single-round accuracy were saved and the model parameters were loaded into the training set. The final determined specific parameters were shown in Table 2.

**Table 2:** Main parameters of the SVM, LSTM, ResNet18, VGG16 and AlexNet models

Model	Model parameters	Value	
SVM	The Kernel Function		Polynomial Kernel
	Cache_size		200
	Tol (Tolerance Used in the IterativeAlgorithm)		10^− 3^
	Max_iter		-1
	C (Regularization Parameter)		1
LSTM	Normalize		L2
	Optimizer		Adam (Adaptive moment estimation)
	Activation Function		Tanh (TanHyperbolic)
	NumHiddenUnits		20
	Learning Rate		0.001
	Epochs		40
	Batch Size		64
	Dropout		0.5
	Verbose		1
ResNet18	Learning Rate		0.001
	Epochs		90
	Batch Size		32
	Activation Function		ReLU
	Normalize		L2
VGG16	Learning Rate		0.001
	Epochs		90
	Batch Size		16
	Activation Function		ReLU
	Normalize		L2
AlexNet	Learning Rate		0.001
	Epochs		90
	Batch Size		16
	Activation Function		ReLU
	Normalize		L2

#### Test environment and model evaluation

The conditions for processing data in this experiment are as follows. Hardware Processor: Inter Xeon CPU E5-2640 V4 @ 2.4GHZ 2.40GHZ (two processors); RAM: 128 GB; Software environment: CUDA Toolkit 10.1; CUDN V7.6.0; MATLAB 2020; Python 3.8; Pytorch-GPU 1.6.0; Operating system: Windows 10.

To evaluate the performance of the model, the four indexes, Accuracy, Precision, Recall, and F1-score were used. To evaluate the overall ability of the tea coal disease classification model, the Accuracy index was used, which refers to the proportion of correctly identified samples; the Precision referred to the ratio of the number of correctly identified tea coal disease samples to the total number of identified tea coal disease samples; the Recall was the ratio of the number of correctly identified tea coal disease samples to the total number of tea coal disease samples; and the F1-score was the attempt to do An evaluation index of the coordination between precision and recall. The specific calculation formula was shown in Eq. (2)(3)(4)(5).

2 $$Accuracy=\frac{TP+TN}{TP+FP+TN+FN}$$

3 $$Precision=\frac{TP}{TP+FP}$$

4 $$Recall=\frac{TP}{TP+FN}$$

5 $$F1-score=\frac{2*precision*recall}{precision+recall}$$

The “TP” (True Positive) indicated the number of samples correctly identified as tea coal disease. The “FN” (False Negative) is the number of samples not identified as tea coal disease. The “FP” (False Positive) is the number of samples incorrectly identified as tea coal disease. The “TN” (True Negative) is the number of samples correctly identified as health samples [29].

## Results and analysis

### Data preprocessing

The RGB image samples were flipped 90°, 180°, 270°, vertically, and horizontally to expand the sample data by a factor of 5. Furthermore, the sample size of RGB images was divided into the training set, the testing set and the validation set in the ratio of 3:1:1.

**Fig. 5:**
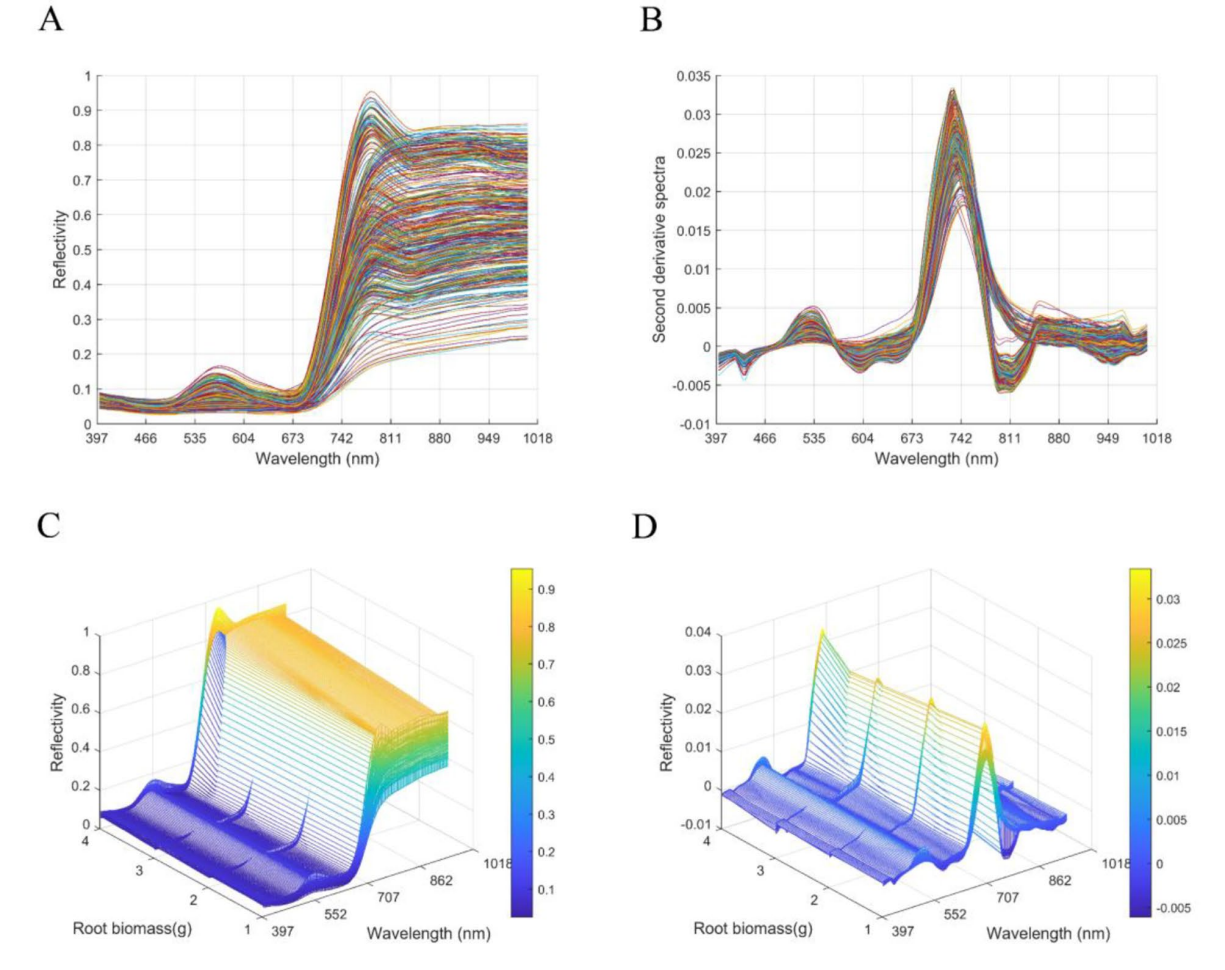
Original spectra and Spectra after preprocessing. (**A**) 2-D Original spectra; (**B**) 2-D Spectra after preprocessing; (**C**) 3-D Original spectra; (**D**) 3-D Spectra after preprocessing

To illustrate the differences between different disease severity levels, an average original spectrum was plotted for visualization. 2D and 3D images of spectral characteristics of four disease severity levels can be compared. 1,2,3,4 represent mild, moderate, severe, and standard degrees, respectively (Fig. 5A, C). From the spectral features, the overall trend of the average spectral reflectance of the four disease severities in the specified wavelength band was similar, and the difference between the two seasonal samples could be clearly seen from the two-dimensional spectral image features. Still, the overall trend of the two seasonal samples was similar. The spectral features had three inflection points, near 552, 673, and 800 nm wavelengths, respectively. The reflectance of the four disease severities showed an increasing trend in the wavelength range of 397–552 nm, and a decreasing trend in the wavelength range of 552–673 nm. In the wavelength range of 673–800 nm, the reflectance of all four disease severities increased exponentially, and a significant difference could be observed in the reflectance of reaching the inflection point with increasing disease severity, which increased between 0.7 and 0.95.

The SNV, 2-D, and S-G algorithms were used to preprocess the hyperspectral data to avoid the effects of unwanted and interfering signals and random noise and to improve the spectral resolution (Fig. 5B, D). The results showed that compared with the original spectra, the spectral curves after combined pretreatment with SNV, 2-D, and S-G were more stable, with more prominent peaks and valleys, and the accuracy and reliability of the model were higher. Similarly, from the perspective of spectral characteristics, the overall trend in spectral reflectance was similar for the four disease severities. The variation was significant in the wavelength range of 673–811 nm, with the reflectance peaking near the wavelength of 742 nm. In addition, there were significant differences between the spectra of the four disease severities, which could be clearly distinguished.

### Feature band selection for spectral data

Although there is a high correlation between hyperspectral bands, as bands and samples increase, problems such as band collinearity and data redundancy may arise [44]. Therefore, we conduct feature band screening on spectral data to reduce the impact of unrelated bands, reduce the complexity of the model, and improve the accuracy of the model [45–47].

**Fig. 6:**
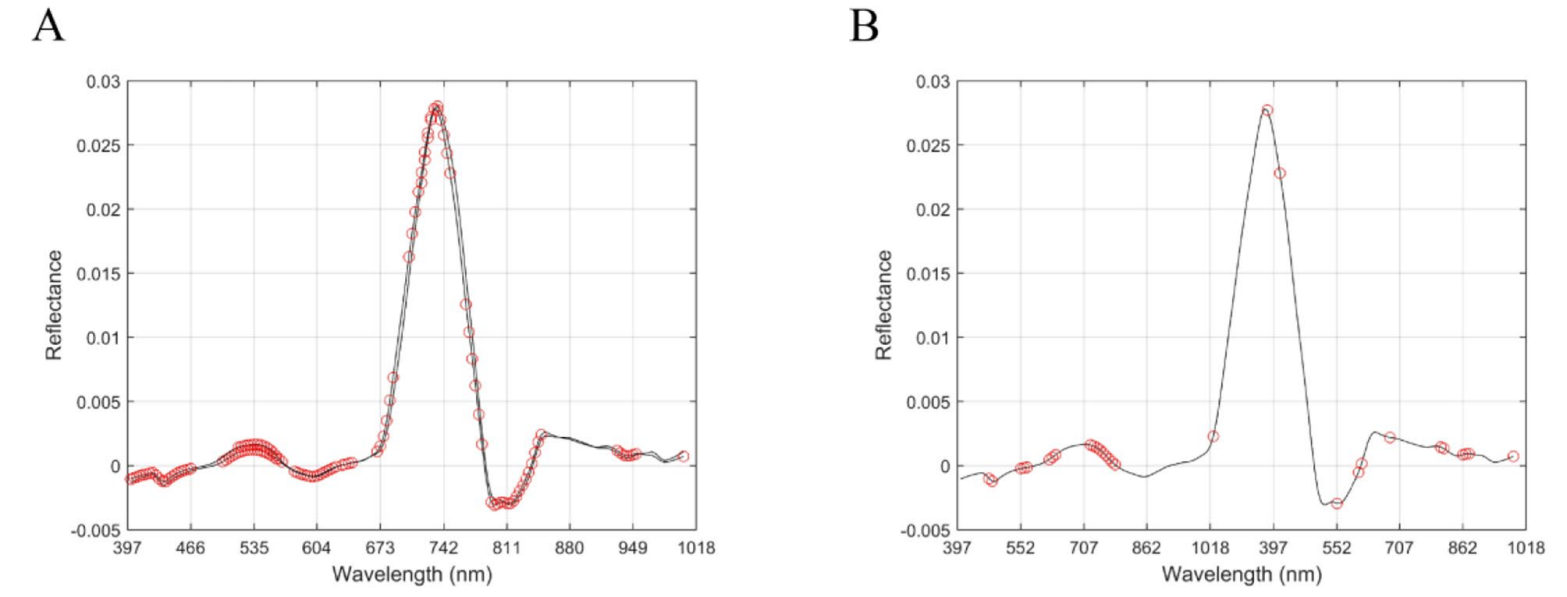
Distribution of characteristic bands. (**A**) UVE; (**B**) CARS.

This study used UVE and CARS algorithms to filter feature bands (Fig. 6; Table 3). The results showed that among the feature band screening methods, the number of feature bands screened by UVE was the highest, with 91 bands, while the number of feature bands screened by CARS was the lowest, with 30 bands.

### Overall accuracy of the model

To compare the performance of RGB imaging technology and hyperspectral imaging technology under different models, the various models of the two imaging techniques were tested under the same test environment and data set, respectively (Table 4). As the network depth increases, the training loss decreases and the network performance was more optimized. Among them, the classification accuracy of ResNet model enhanced by wavelet transform (WT-ResNet)was 70% under RGB imaging technique, which was the highest among the six models, and the enhancement process using the wavelet transform could significantly improve the model accuracy by 5-10%, which was consistent with the results of our team’s previous studies, but all of them were lower than the accuracy of hyperspectral imaging technique, which indicated that hyperspectral imaging technique was significantly better than RGB imaging technique. Under the hyperspectral imaging technique, the classification accuracy of the CARS-LSTM model was 95%, which was the highest among the six models. The model accuracy of LSTM under the same feature band screening method was significantly better than SVM, indicating that the deep learning approach was significantly better than the traditional machine learning approach, The classification accuracies of different feature band filtering methods under the same modeling algorithm (SVM and LSTM) were CARS > NONE > UVE method, indicating that the CARS method filters the feature bands better than the UVE method filters the feature bands and the full band method.

**Table 3:** Bands screening results

Screening Method	Number of Bands	Characteristic Bands (nm)
UVE	91	397–458,507–554,618–628,655–672,689–734,752–769,780–840,924–946,1001
CARS	30	426–429,458–465,488–494,531–557,662,721,734,798,823–836,858,916–920,942–949,1001

### Comparison of the performance of different network models for grading different disease

**Fig. 7:**
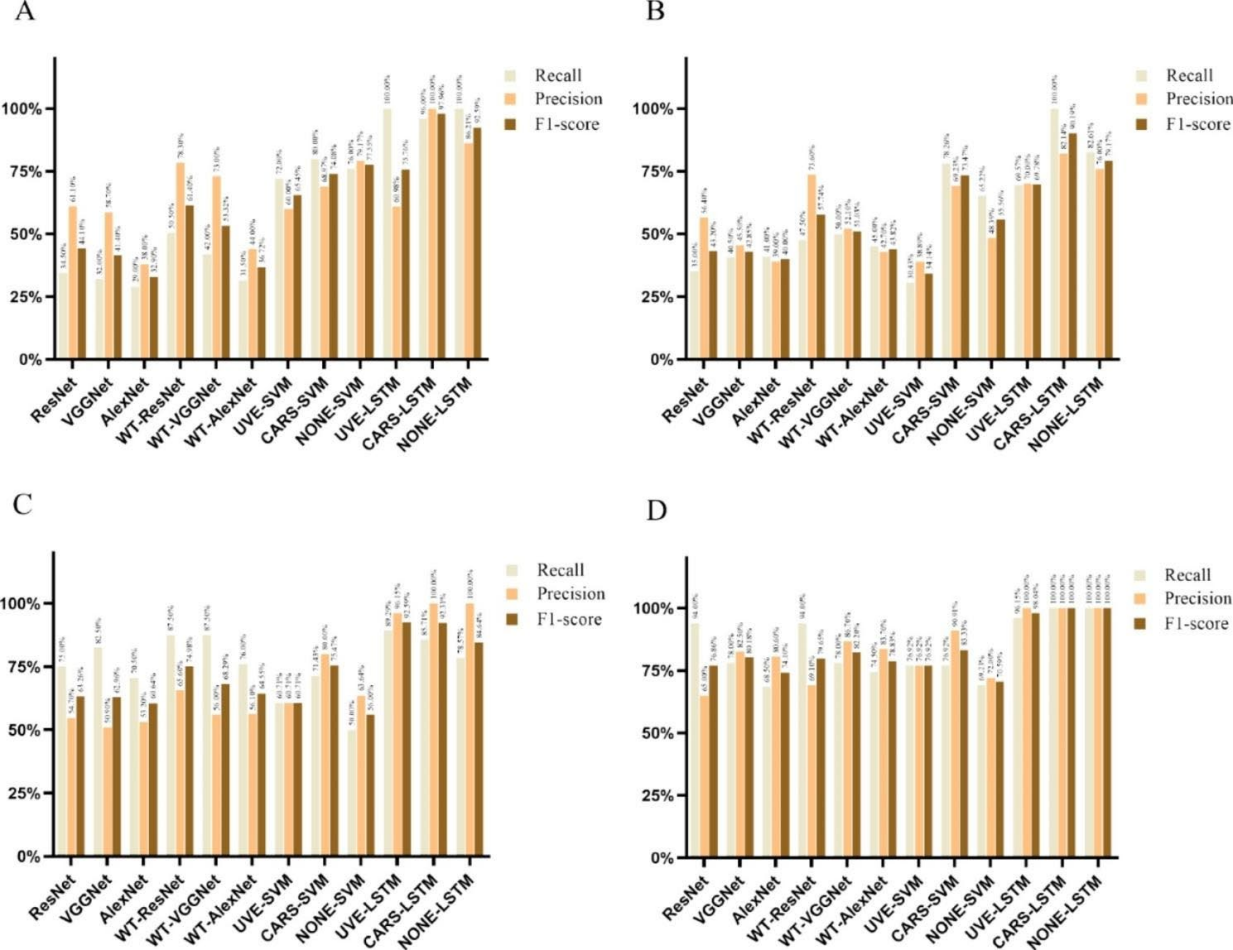
Evaluation results of different network models for different disease degree grading. (**A**) mild degree; (**B**) moderate degree; (**C**) severe degree; (**D**) standard

To further compare the classification performance of RGB imaging and hyperspectral imaging techniques under different models, three evaluation indexes, Recall, Precision, and F1-score, were used to evaluate twelve models (Fig. 7). The results showed that among the six models under the RGB image technique, the models enhanced by wavelet transform were significantly improved in each metric, and the WT-ResNet18 model with 70% precision performed better under the RGB image technique. Among the six models under the hyperspectral image technique, the CARS-LSTM model with 95% accuracy performed better. Overall, for classifications 2 and 4, the CARS-LSTM model had the highest three evaluation indexes. For the classification of 1, the CARS-LSTM model had the highest two evaluation indexes of precision and F1-score, the Recall evaluation index was 96% and the 100% difference between the two models of UVE-LSTM and NONE-LSTM was not significant (only 4%). For the classification of 3, the CARS-LSTM model had the highest precision evaluation index, and the other two evaluation indexes Recall and F1-score differed little from the UVE-LSTM model (only 3.58% and 0.28%). This might be related to the disease characteristics of 1 and 3, where the network with relatively more feature band screening performed better. In general, the CARS-LSTM model had the best overall performance in grading the disease severity of tea coal disease.

### Confusion matrix

**Fig. 8:**
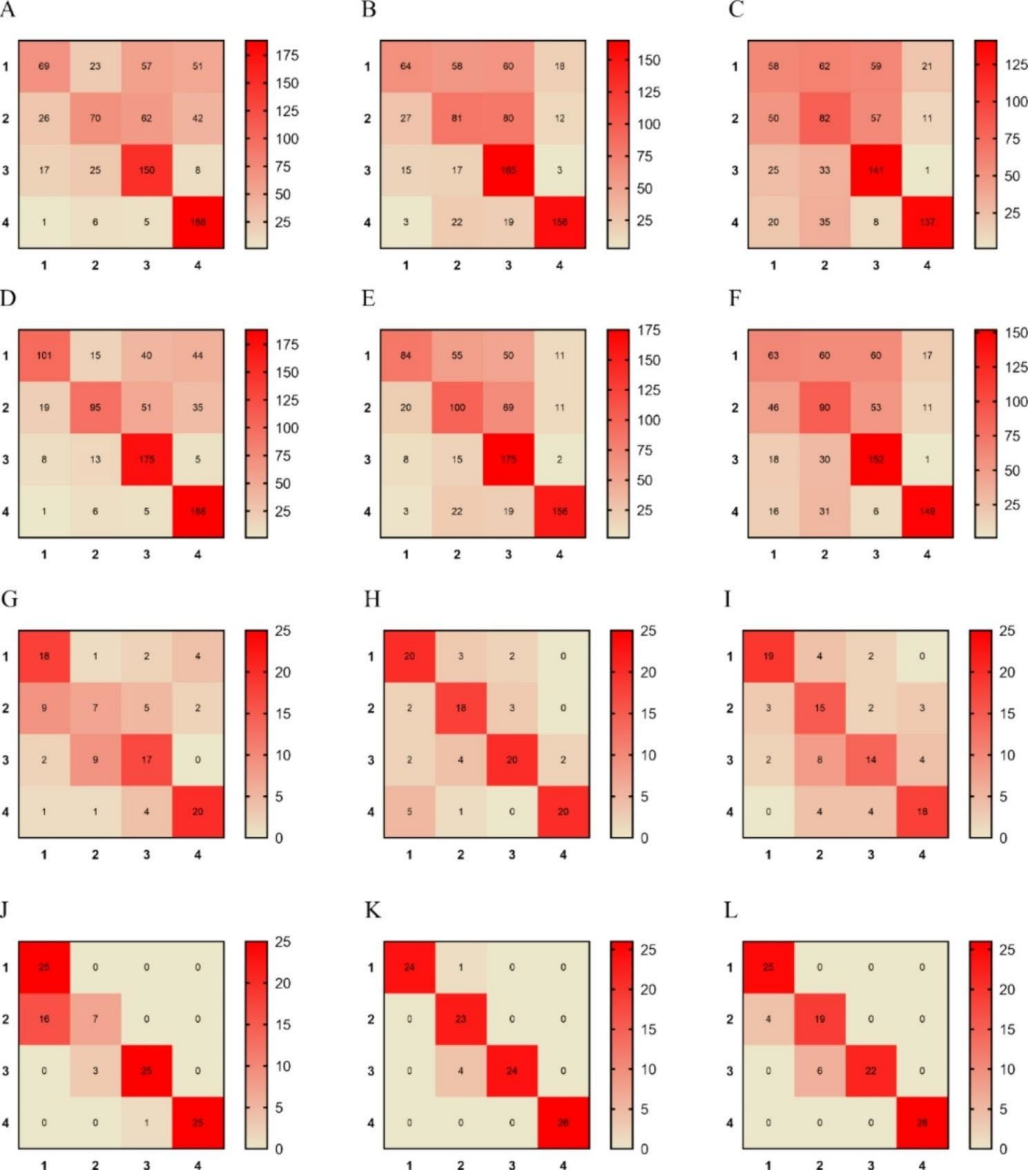
Confusion matrix for different network models. (**A**) ResNet; (**B**) VGGNet; (**C**) AlexNet; (**D**) WT-ResNet; (**E**) WT-VGGNet; (**F**) WT-AlexNet; (**G**) UVE-SVM; (**H**) CARS-SVM; (**I**) NONE-SVM; (**J**) UVE-LSTM; (**K**) CARS-LSTM; (**L**) NONE-LSTM

The confusion matrices were used to observe the misclassification between the four categories of disease severity (Fig. 8). The results showed that none of the six models for RGB imaging (Fig. 8A, B, C, D, E and F) could distinguish well between 1 and 2, despite significant improvements in the wavelet transform enhancement processing method. About 65% of 1s is misclassified as the other three categories and about 60% of 2s is misclassified as the other three categories. There were two possible reasons for this situation. One was that the features of the two degrees of mild and moderate disease were too similar for the model to recognize; secondly, the RGB images, with only three bands of red, green, and blue, were much less accurate than the hyperspectral images with 176 bands in recognizing the degree of disease, leading to the confusion of the features of these two degrees of disease. Therefore, in the subsequent study, we could increase the number of images and collect images with distinct texture features to further optimize the model. For the six models of hyperspectral imaging (Fig. 8G, H, I, J, K, and L), the misclassification was much less than that of the RGB imaging technique model. The misclassification of the LSTM model under hyperspectral imaging was less than that of the SVM model, which indicated that the deep learning approach was significantly better than the traditional machine learning approach. Finally, we found that the CARS-LSTM model can classify accurately with the least misclassification, and the Accuracy of this model is 95%. The results indicated that the CARS-LSTM model has good robustness and can accurately classify tea coal disease.

## Discussion

In this study, we compared the accuracy of six tea coal disease classification models based on RGB imaging technology and six tea coal disease classification models based on hyperspectral imaging technology. The results showed that the CARS-LSTM model gave the best results with an accuracy of 95%. This indicated that the CARS-LSTM model could classify complex and similar disease levels. We analyzed the reasons why the CARS-LSTM model outperforms other models. First, the captured hyperspectral images had 176 bands, which were much more informative than the 3 bands of red, green, and blue in RGB images; second, the model used a deep learning method of LSTM; finally, the CARS method filtered out fewer feature bands than UVE, eliminating redundant feature bands.

Hyperspectral imaging technology was superior to RGB imaging technology. RGB imaging had a limited capacity for recognition but was relatively inexpensive, whereas hyperspectral imaging offered great information and accuracy but was more expensive. We adopted and compared RGB and hyperspectral imaging approaches, respectively, for the purpose of tea coal disease classification. We also compared deep learning and machine learning algorithms, and the findings showed that hyperspectral imaging techniques achieved better results in disease classification. This was a great innovation compared to the single imaging technique used by previous authors. In the study by Yuan et al. [48], hyperspectral imaging was used to detect anthracnose in tea trees, and the results showed an overall accuracy of 98% at the leaf level and 94% at the pixel level in identifying the disease. In the study by Alves et al. [49], the visible spectral regions of the symptomatic leaves of five diseases, including soybean rust (SBR), Calonectria leaf blight (CLB), wheat leaf blight (WLB), Nicotiana tabacum-Xylella fastidiosa (NtXf), and potato late blight (PLB), were studied using RGB images. The results showed that the SBR, CLB, and WLB models achieved high prediction accuracy (> 97%) on the testing set. the prediction accuracy of both NtXf and PLB models was below 90%. In this case, the highest accuracy rate of 95% was achieved using hyperspectral images with a deep learning algorithm, much higher than the highest accuracy rate of 60% achieved using RGB images with a deep learning algorithm the reason behind this captured hyperspectral images have 176 bands, which are much more informative than the three bands of red, green, and blue of RGB images. Therefore, the results also highlight the importance of high-throughput acquisition methods.

Moving forward, the model accuracy was higher for the CARS algorithm screening fewer feature bands. In contrast, the UVE algorithm for the models screening more feature bands and the full band model was less accurate. Various reports have shown that selecting some significant spectral variables represented better prediction results than spectra containing redundant variables [50, 51]. In the study by Yang et al., they observed that the characteristic wavelength model produced after screening performed better than the complete wavelength model. This was established by comparing the spectral data processed and unprocessed by the model [52]. Therefore, the selection of characteristic wavelengths was an essential step in processing a large amount of hyperspectral image spectral data, and by this step, the amount of data could be reduced, and prediction models with more vital generalization ability could be obtained.

Deep learning algorithms outperformed machine learning algorithms. The deep learning algorithms of ResNet18, VGGNet16 and AlexNet were used to model the RGB image data, and the algorithms of machine learning of SVM and the deep learning algorithm of LSTM were used to model the hyperspectral image data (Table 4). The results demonstrated that the deep learning models performed better, indicating that the extracted feature strips covered the feature information of the four disease severities. Among them, the CARS-LSTM model exhibited the highest accuracy, with a 95% accuracy. It was consistent with prior research findings. In our team’s study by Li et al. [29], five models, F-RNet, ResNet18, VGG16, AlexNet and SVM, were developed to identify three tea pests and disease symptoms. The results showed that the deep learning models such as ResNet18, VGG16 and AlexNet had 82%, 80% and 73% accuracy, respectively, which were significantly greater than the SVM machine learning model with 65% accuracy. In the study by Goluguri, Devi and Srinivasan [53], three models, DCNN-LSTM, DCNN-SVM, and DCNN-ANN, were developed for rice disease identification. The results showed that the EAFSO (efficient artificial fish swarm optimization) joint DCNN-LSTM deep learning model identified rice diseases with 97.5% accuracy, much better than the machine learning models of DCNN-SVM and DCNN-ANN. In this case, the CARS feature band filtering method with the deep learning algorithm achieved the highest 95% accuracy, which is much higher than other algorithms. This is because it is better to select some representative spectra than those containing redundant variables. Besides, the depth of the model structure of the deep learning algorithm is better than that of the machine algorithm. Hence, the results also highlight the importance of the CARS algorithm and deep learning.

**Table 4:** Accuracy of two imaging techniques with different models for testing disease Classification

Image type	Model	Accuracy (%)
RGB image	ResNet18	60
	VGGNet16	58
	AlexNet	52
	WT-ResNet18	70
	WT-VGGNet16	64
	WT-AlexNet	57
Hyperspectral image	UVE-LSTM	80
	CARS-LSTM	95
	NONE-LSTM	90
	UVE-SVM	61
	CARS-SVM	77
	NONE-SVM	65

Considering the practical application, it was almost impossible to apply the hyperspectral imaging system to real-time disease identification and classification in tea plantations due to its high cost and lengthy processing cycle. Consequently, we planned to combine theory and practice to solve the problems in agricultural production by establishing a disease detection platform and realizing real-time data reception for rapid judgment through manual remote control, pending further research. This study exclusively obtained samples of tea coal disease from a singular geographic region during two distinct seasons. In future research, we can collect tea coal disease samples from more seasons and more geographic areas to increase the data set, thus enhancing the generalization of the model and studying the effects of tea coal disease seasons and geographic areas on the model performance in more depth.

## Conclusion

This study established a classification model of tea coal disease based on RGB and hyperspectral imaging technology to classify the severity of tea coal disease. RGB and hyperspectral images of leaves with different degrees of tea coal disease were collected. Respectively using deep learning algorithms such as ResNet18, VGG16, and AlexNet for RGB image data to establish a classification model for the severity of tea coal disease; Three methods, SNV, 2-D, and S-G, were used for spectral preprocessing of hyperspectral image data. Two methods, UVE and CARS, were used to screen characteristic bands. SVM and LSTM algorithms were used to establish a classification model for the severity of tea coal disease.

The results indicated that the residual network enhanced by wavelet transform outperformed other networks in the RGB imaging technology classification model. Among the hyperspectral feature band screening methods, the CARS was superior to the UVE; among the deep learning and machine learning algorithms, the LSTM algorithm was superior to the SVM algorithm. Both RGB and hyperspectral could be used to classify tea coal disease. The RGB optimal classification model was WT-ResNet18, and the hyperspectral optimal classification model was CARS-LSTM. At the same time, the model based on hyperspectral imaging was superior to the model based on RGB imaging.

In summary, we selected the CARS-LSTM classification model for the severity of tea coal disease, with an accuracy of 95%. This study revealed the classification potential of tea coal disease based on RGB and hyperspectral imaging, which can provide an accurate, non-destructive, and efficient classification method for tea coal disease monitoring.

## Data Availability

Not applicable.

## References

[1] Lv L, Zhao F. Identification of Tea Plant Diseases and Pests and Green Prevention and Control. Zhongyuan Farmers’ Publishing House; 2010.

[2] Zhou T, Yu J, Hu X. Primary Color Map of Tea Pest Control. Zhejiang Science and Technology Press; 2010.

[3] Bock CH, Poole GH, Parker PE, Gottwald TR (2010). Plant Disease Severity estimated visually, by Digital Photography and Image Analysis, and by Hyperspectral Imaging. CRC Crit Rev Plant Sci.

[4] Martinelli F, Scalenghe R, Davino S, Panno S, Scuderi G, Ruisi P (2014). Advanced methods of plant disease detection. A review. Agron Sustain Dev.

[5] Ali MM, Bachik NA, Muhadi NA, Tuan Yusof TN, Gomes C. Non-destructive techniques of detecting plant diseases: a review. Physiol Mol Plant Pathol. 2019;108.

[6] Sankaran S, Mishra A, Ehsani R, Davis C (2010). A review of advanced techniques for detecting plant diseases. Comput Electron Agric.

[7] Oerke EC, Herzog K, Toepfer R (2016). Hyperspectral phenotyping of the reaction of grapevine genotypes to Plasmopara viticola. J Exp Bot.

[8] Jayapal PK, Park E, Faqeerzada MA, Kim Y-S, Kim H, Baek I et al. Analysis of RGB Plant images to identify Root rot Disease in korean ginseng plants using deep learning. Appl Sci. 2022;12(5).

[9] Amarasingam N, Gonzalez F, Salgadoe ASA, Sandino J, Powell K. Detection of White Leaf Disease in sugarcane crops using UAV-Derived RGB Imagery with existing Deep Learning Models. Remote Sens. 2022;14(23).

[10] Hallau L, Neumann M, Klatt B, Kleinhenz B, Klein T, Kuhn C (2018). Automated identification of sugar beet diseases using smartphones. Plant Pathol.

[11] Sie EK, Oteng-Frimpong R, Kassim YB, Puozaa DK, Adjebeng-Danquah J, Masawudu AR (2022). RGB-image method enables indirect selection for leaf spot resistance and yield estimation in a groundnut breeding program in Western Africa. Front Plant Sci.

[12] Memon MS, Kumar P, Iqbal R. Meta Deep learn Leaf Disease Identification Model for Cotton Crop. Computers. 2022;11(7).

[13] Feng L, Wu B, He Y, Zhang C (2021). Hyperspectral imaging combined with deep transfer learning for Rice Disease Detection. Front Plant Sci.

[14] Zhao J, Fang Y, Chu G, Yan H, Hu L, Huang L. Identification of Leaf-Scale Wheat Powdery Mildew (Blumeria graminis f. sp. Tritici) combining Hyperspectral Imaging and an SVM Classifier. Plants (Basel). 2020;9(8). 10.3390/plants9080936 PMC7464903 32722022

[15] Abdulridha J, Batuman O, Ampatzidis Y. UAV-Based remote sensing technique to detect Citrus Canker Disease utilizing Hyperspectral Imaging and Machine Learning. Remote Sens. 2019;11(11).

[16] Wu G, Fang Y, Jiang Q, Cui M, Li N, Ou Y et al. Early identification of strawberry leaves disease utilizing hyperspectral imaging combing with spectral features, multiple vegetation indices and textural features. Comput Electron Agric. 2023;204.

[17] Lee CC, Koo VC, Lim TS, Lee YP, Abidin H (2022). A multi-layer perceptron-based approach for early detection of BSR disease in oil palm trees using hyperspectral images. Heliyon.

[18] Zhang C, Liu F, Feng XP, He Y, Bao YD, He LW (2017). Comparison and selection of vegetation indices for detection of Sclerotinia Stem rot on oilseed rape leaves using ground-based hyperspectral imaging. Adv Anim Biosci.

[19] Harakannanavar SS, Rudagi JM, Puranikmath VI, Siddiqua A, Pramodhini R (2022). Plant leaf disease detection using computer vision and machine learning algorithms. Global Transitions Proceedings.

[20] Koc A, Odilbekov F, Alamrani M, Henriksson T, Chawade A (2022). Predicting yellow rust in wheat breeding trials by proximal phenotyping and machine learning. Plant Methods.

[21] Dias F, Valente D, Oliveira C, Dariva F, Copati M, Nick C (2023). Remote sensing and machine learning techniques for high throughput phenotyping of late blight-resistant tomato plants in open field trials. Int J Remote Sens.

[22] Gao J, Zhao L, Li J, Deng L, Ni J, Han Z (2021). Aflatoxin rapid detection based on hyperspectral with 1D-convolution neural network in the pixel level. Food Chem.

[23] Sujatha R, Chatterjee JM, Jhanjhi NZ, Brohi SN. Performance of deep learning vs machine learning in plant leaf disease detection. Microprocess Microsyst. 2021;80.

[24] Ma J, Du K, Zheng F, Zhang L, Gong Z, Sun Z (2018). A recognition method for cucumber diseases using leaf symptom images based on deep convolutional neural network. Comput Electron Agric.

[25] Wang G, Sun Y, Wang J (2017). Automatic image-based Plant Disease Severity Estimation using deep learning. Comput Intell Neurosci.

[26] Gao C, Ji X, He Q, Gong Z, Sun H, Wen T et al. Monitoring of wheat Fusarium Head Blight on Spectral and Textural Analysis of UAV Multispectral Imagery. Agriculture. 2023;13(2).

[27] Sood S, Singh H, Jindal S. Rust disease classification using deep learning based Algorithm: the case of wheat. Food Systems Resilience. Sustainable Development; 2022.

[28] Huang Y, Wang D, Liu Y, Zhou H, Sun Y. Measurement of Early Disease Blueberries based on Vis/NIR Hyperspectral Imaging System. Sens (Basel). 2020;20(20). 10.3390/s20205783 PMC7600744 33066056

[29] Li H, Shi H, Du A, Mao Y, Fan K, Wang Y (2022). Symptom recognition of disease and insect damage based on Mask R-CNN, wavelet transform, and F-RNet. Front Plant Sci.

[30] Sun Y, Wang Y, Xiao H, Gu X, Pan L, Tu K (2017). Hyperspectral imaging detection of decayed honey peaches based on their chlorophyll content. Food Chem.

[31] Schafer R (2011). What is a Savitzky-Golay Filter? [Lecture Notes]. IEEE Signal Process Mag.

[32] Kong W, Liu F, Zhang C, Bao Y, Yu J, He Y (2014). Fast detection of peroxidase (POD) activity in tomato leaves which infected with Botrytis cinerea using hyperspectral imaging. Spectrochim Acta A Mol Biomol Spectrosc.

[33] Mao Y, Li H, Wang Y, Fan K, Shen J, Zhang J (2023). Low temperature response index for monitoring freezing injury of tea plant. Front Plant Sci.

[34] Qing H, He-ru X, Jiang-ping L, Mei-chen L, Peng-wei H (2022). De-gang S. Spectral Selection Method based on ant colony-genetic algorithm. Spectrosc Spectr Anal.

[35] Shu M, Shen M, Zuo J, Yin P, Wang M, Xie Z (2021). The application of UAV-Based hyperspectral imaging to Estimate crop traits in maize inbred lines. Plant Phenomics.

[36] Li Z, Wang J, Xiong Y, Li Z, Feng S (2016). The determination of the fatty acid content of sea buckthorn seed oil using near infrared spectroscopy and variable selection methods for multivariate calibration. Vib Spectrosc.

[37] Wu D, Chen X, Zhu X, Guan X, Wu G. Uninformative variable elimination for improvement of successive projections algorithm on spectral multivariable selection with different calibration algorithms for the rapid and non-destructive determination of protein content in dried laver. Anal Methods. 2011;3(8).

[38] Yuan R, Liu G, He J, Wan G, Fan N, Li Y et al. Classification of Lingwu long jujube internal bruise over time based on visible near-infrared hyperspectral imaging combined with partial least squares-discriminant analysis. Comput Electron Agric. 2021;182.

[39] Boser BE, Guyon IM, Vapnik VN, editors. A training algorithm for optimal margin classifiers. Proceedings of the fifth annual workshop on Computational learning theory; 1992.

[40] Zhang S, Huang H, Huang Y, Cheng D, Huang J. A GA and SVM classification model for Pine Wilt Disease Detection using UAV-Based Hyperspectral Imagery. Appl Sci. 2022;12(13).

[41] Guo Y, Qu F, Yu Z, Yu Q (2020). Deep LSTM with guided filter for hyperspectral image classification. Comput Inform.

[42] Li H, Mao Y, Wang Y, Fan K, Shi H, Sun L et al. Environ Simul Model Rapid Prediction Tea Seedl Growth Agron. 2022;12(12).

[43] Turkoglu M, Hanbay D, Sengur A (2019). Multi-model LSTM-based convolutional neural networks for detection of apple diseases and pests. J Ambient Intell Humaniz Comput.

[44] Wu D, Nie P, He Y, Bao Y (2011). Determination of Calcium Content in Powdered milk using Near and Mid-Infrared Spectroscopy with Variable Selection and Chemometrics. Food Bioprocess Technol.

[45] Sarathjith MC, Das BS, Wani SP, Sahrawat KL (2016). Variable indicators for optimum wavelength selection in diffuse reflectance spectroscopy of soils. Geoderma.

[46] Xu S, Zhao Y, Wang M, Shi X (2017). Determination of rice root density from Vis–NIR spectroscopy by support vector machine regression and spectral variable selection techniques. CATENA.

[47] Mao Y, Li H, Wang Y, Fan K, Song Y, Han X et al. Prediction of tea polyphenols, free amino acids and Caffeine Content in Tea Leaves during Wilting and Fermentation using Hyperspectral Imaging. Foods. 2022;11(16). 10.3390/foods11162537 PMC9407140 36010536

[48] Yuan L, Yan P, Han W, Huang Y, Wang B, Zhang J et al. Detection of anthracnose in tea plants based on hyperspectral imaging. Comput Electron Agric. 2019;167.

[49] Alves KS, Guimarães M, Ascari JP, Queiroz MF, Alfenas RF, Mizubuti ESG (2021). RGB-based phenotyping of foliar disease severity under controlled conditions. Trop Plant Pathol.

[50] Wu D, Chen X, Shi P, Wang S, Feng F, He Y (2009). Determination of alpha-linolenic acid and linoleic acid in edible oils using near-infrared spectroscopy improved by wavelet transform and uninformative variable elimination. Anal Chim Acta.

[51] ElMasry G, Iqbal A, Sun D-W, Allen P, Ward P (2011). Quality classification of cooked, sliced turkey hams using NIR hyperspectral imaging system. J Food Eng.

[52] Yang C, Zhao Y, An T, Liu Z, Jiang Y, Li Y et al. Quantitative prediction and visualization of key physical and chemical components in black tea fermentation using hyperspectral imaging. Lwt. 2021;141.

[53] Goluguri NVRR, Devi KS, Srinivasan P (2020). Rice-net: an efficient artificial fish swarm optimization applied deep convolutional neural network model for identifying the Oryza sativa diseases. Neural Comput Appl.

